# Intranodal palisaded myofibroblastoma shows a unique epigenetic profile—first molecular study of their epigenetic and copy number variation profile

**DOI:** 10.1007/s00428-025-04170-x

**Published:** 2025-07-18

**Authors:** Sandra Leisz, Maximilian Scheer, Uwe Hildebrandt, Merle Wiegers, Christian Strauss, Christian Scheller, Thomas Mentzel, Andreas von Deimling, Anja Harder

**Affiliations:** 1 Department of Neurosurgery, Medical Faculty, Martin Luther University Halle-Wittenberg, Halle (Saale), Germany; 2 Institute of Pathology, Harz Clinic, Quedlinburg, Germany; 3 MVZ Dermatopathology, Friedrichshafen, Germany; 4 Department and CCU Neuropathology, University Hospital Heidelberg and German Cancer Center (DKFZ), Heidelberg, Germany; 5 CURE-NF Research Group, Medical Faculty, Martin Luther University Halle-Wittenberg, Halle (Saale), Germany; 6 Institute of Neuropathology, University Hospital Münster, Münster, Germany

**Keywords:** Myofibroblastoma, Intranodal palisaded myofibroblastoma, Methylome, Copy number variation profile, Amianthoid fiber, Schwannoma, Molecular, Methylation class

## Abstract

Intranodal palisaded myofibroblastomas with amianthoid fibers (IPM) are rare mesenchymal neoplasms showing a myofibroblastic differentiation. Histopathologically, they might be difficult to distinguish from schwannoma or other neoplasia with spindle cell morphology, especially on limited biopsies. *CTNNB1* gene variants have been detected in at least 50% of tumors. In this study, we determined the methylation profile including the copy number variation profile in a series of six patients. These analyses enabled genes with the highest gains or losses compared to myoblasts and fibroblasts to be identified. We identified a new methylation cluster that is not included in the Heidelberg Sarcoma Classifier so far. Furthermore, significantly differentially hypo- and hypermethylated genes compared to normal myoblasts and fibroblasts were detected in all samples, e.g., ARID5A, MIB2, TRIM58, and others were > 17-fold hypomethylated, while NEDD4, RUNX1, SLC8A1, and others were > 75-fold hypermethylated. Additionally, when combining positive ß-catenin expression and sequencing results, the aberrant/mutant *CTNNB1* gene was shown in three tumors (75% of analyzed cases) in this IPM series. The present data provides additional support/adjunct to establish the rare diagnosis of intranodal palisaded myofibroblastomas with amianthoid fibers by molecular testing in diagnostically challenging cases.

**Supplementary Information:**

The online version contains supplementary material available at 10.1007/s00428-025-04170-x.

## Introduction

Intranodal palisaded myofibroblastoma with amianthoid fibers (IPM) are rare slowly growing mesenchymal tumors with a good prognosis. Usually, the tumors are asymptomatic and painless. The diagnosis can be confirmed using fine needle aspiration due to their superficial localizations. IPM demonstrate a differentiation reminiscent of myofibroblasts [1–3]. The prominent collagen fiber compartment in IPM is reminiscent of amianthus, an older term for asbestos fibers and therefore name giving. However, electron microscopy demonstrated those structures to consist of collagen fibers with a width of 80–150 nm, forming thick bundles [3, 4]. Tumors arising within lymph nodes have been reported. In a larger series, IPM mainly occurred between ages 45 and 55 and more frequently in male patients.

The diagnosis is usually made by conventional histological and immunohistochemical analysis. Abnormal expression of β-catenin and cyclin D1 was described and related to a hotspot missense pathogenic variant within the catenin beta-1 (*CTNNB1*) gene (mutation of exon 3 is reported in 71% of IPM cases) [5, 6]. IPM can be mistaken for other soft tissue tumors, such as schwannoma, desmoid fibromatosis, sarcomas, or melanomas with spindle cell morphology at first glance [3, 7, 8]. Formerly, they were called “intranodal hemorrhagic spindle cell tumor with amianthoid fibers” due to their high susceptibility for intratumorally bleeding and also “schwannoma of the lymph node” due to their localization within inguinal lymph nodes.

As the ß-catenin expression is the only recurrent molecular event due to *CTNNB1* variants and is also detected in other mesenchymal neoplasms, we aimed to establish a molecular tumor profile of IPM. Knowledge on the molecular landscape might help to distinguish morphological mimics in case of sparse material or of unusual localization [9, 10]. To define genetic criteria for IPM, we performed a detailed molecular analysis in our case series and sought to characterize the methylation and copy number profiles of IPM.

## Materials and methods

### Patients, histopathology, and immunohistochemistry

Cases were retrieved from the Institute of Pathology at the Harz Clinic of Quedlinburg and the diagnostic Centre of Dermatopathology in Friedrichshafen, Germany. Formalin-fixed paraffin-embedded (FFPE) tumor tissue material from an inguinal lymph node was provided for histopathological, immunohistochemical, and molecular analysis. Six patients with diagnosed IPM were included. The age at onset ranged from 42 to 82 years (Table S1). The tumor samples were collected in a period from 2010 to 2022.

All histological sections of the reported cases were stained with hematoxylin and eosin (H&E), some with periodic acid Schiff (PAS) reaction and Congo red. Immunohistochemical analyses were performed as reported in Table S2.

### DNA isolation

IPM tumor areas were reviewed and marked by a neuropathologist (AH). Tumor tissue was removed from FFPE slides. Tumor content was estimated to be above 95% of all cells. Genomic DNA was extracted using the QIAamp DNA FFPE Advanced Kit (Qiagen, Hilden, Germany). The DNA concentration and purity were determined by measurement of the optical density at 260 nm, 280 nm, and 310 nm with a plate reader (Tecan Infinite M200 Pro, Tecan, Männedorf, Switzerland).

### Microarray-based methylation profiling (EPIC 850 k array) and data evaluation

Molecular analysis was performed using Infinium Methylation EPIC ‘850 K’ BeadChip Array (Illumina, San Diego, USA) using FFPE-derived genomic tumor DNA. Raw data of the Methylation EPIC ‘850 K’ BeadChip array were analyzed using the GenomeStudio software (v2011.1, 2022, Methylation module, Illumina, San Diego, USA). The prepared data were further analyzed by python scripts, which enabled the utilization of the common libraries Plotly [11], Matplotlib [12], NumPy [13], and pandas [14]. For reference, we used datasets from human fibroblasts and myoblasts also being examined by the Infinium Methylation EPIC-Chip (GEO Series accession number GSE213427 (https://www.ncbi.nlm.nih.gov/geo/query/acc.cgi?acc=GSE213427) and compared those with our IPM datasets. To compare the datasets, Venn diagrams were created, and the overlap of all samples was considered for evaluation and analysis.

Information on copy number variation (CNV) was calculated by extracting the copy numbers from the methylation array using the Bioconductor package minfi [13–15].

For this purpose, the same reference data were used as previously for the methylation data. The overlap of all samples was considered for analysis. The cut-off gains and losses were set to > +/− 0.4. Since the samples from patients C and D showed increased DNA degradation, they were excluded from the comparison of CNV data, sequencing, and methylation.

For visualization and comparison of the methylome using Uniform Manifold Approximation and Projection for Dimension Reduction (UMAP) clustering, we used the Epigenomic Digital Pathology (EpiDiP) analysis version 4.2 GPU (developed by J. Hench, Department of Neuropathology, Institute for Medical Genetics and Pathology, University Hospital Basel, Switzerland). In addition, the Heidelberg classifier (brain classifier version 12.8, last assessed March 13, 2024) and the EpiDip software (version 4.2 GPU, last assessed March 13, 2024) were used to create CNV profiles. Furthermore, the Heidelberg sarcoma classifier (version 12.3) was employed to investigate alignment to a methylation class in the reference set.

### Sanger sequencing of CTNNB1 exon 3

Exon 3 of *CTNNB1* gene was amplified from DNA of samples A, B, E, and F. The primer and PCR conditions used are listed in Table S3. The PCR product was cloned in the pCR2.1 TOPO vector (Invitrogen, Thermo Fisher Scientific, Waltham, USA). Then, the cloned *CTNNB1* exon 3 was analyzed using Sanger sequencing (Eurofins, Ebersberg, Germany). Sequences were aligned to the reference sequences NM_001904.4 (NCBI, Bethesda, USA).

## Results

### Typical histopathological and immunohistochemical features, *CTNNB1 *sequencing

**Fig. 1:**
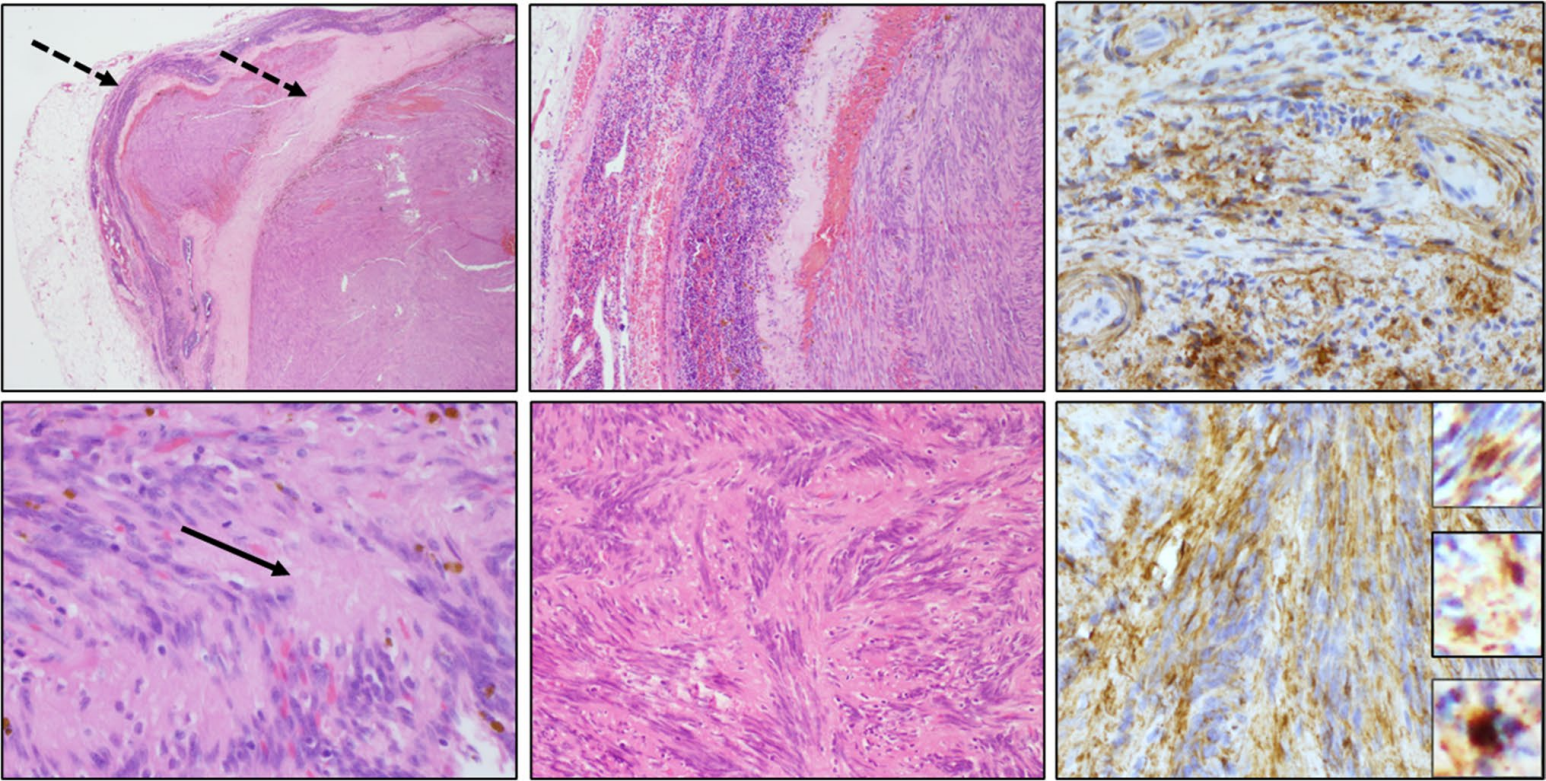
Representative histological growth patterns of an intranodal palisaded myofibroblastoma (IPM) with amianthoid fibers (arrow), a capsule and pseudocapsule (dotted arrows), as well as hemorrhages (index case A). Spindle cells forming fascicles (HE staining, left and middle columns) are immunopositive for smooth muscle actin (upper right) and ß-catenin (below right, three inserts demonstrate positive nuclei from different tumor regions)

Detailed information was available for patient A, a 67-year-old female who presented with a left-sided inguinal nodal lesion suspicious of lymphoma. Additionally, the patient was diagnosed with fibroadenoma of the left breast. At presentation, suspect other lymph nodes were not detected. The removed inguinal lesion measuring 25 × 20 × 20 mm was grossly well demarcated and showed a thin capsule and coarse, elastic, and yellowish cut surface as well as dark bleeding areas. Histopathology showed a typical growth pattern of IPM (Fig. 1) with amianthoid fibers. Eosinophilic spindle cells formed fascicles, and hemorrhage and collagen fibers were present. The presence of typical amianthoid bodies could be detected by the use of Congo red staining, in addition to polarization. Mitotic activity was below 1% (Ki-67 index). By immunohistochemical analysis, spindle cells were positive for actin, smooth muscle actin (SMA), cyclin D1, and ß-catenin, but negative for CD34, EMA, cytokeratin AE1/3, S100, SOX10, CD31, D 2–40, CD117, desmin, h-caldesmon, and neurofilament.

The other five tumors fulfilled the criteria for the diagnosis of IPM. Clinical data and some stainings were not available. These spindle cell tumors showed palisades of cells and fascicles. Some fresh and older hemorrhages were often evident. Both thin and thick collagen fibers were usually present. Typical amianthoid bodies were detectable but varied in their amount. Nuclei had a round to oval or even elongated shape. There was neither suspicious mitotic activity nor signs of atypia. Necrosis or other signs of malignancy were not detected. By immunohistochemical analysis, the spindle cells were positive for smooth muscle actin in all tumors. Expression of CD34, S100, and desmin was not observed (compare Table S2), whereas collagen type IV was seen in three cases (the other two cases were not analyzed for collagen type IV). Nuclear ß-catenin expression was not observed; nevertheless, we detected mutations in two out of three analyzed cases (Table 1, Figure S1). Combining positive ß-catenin expression in case A and sequencing results of three cases, proof of aberrant/mutant *CTNNB1* was shown in 75% of this IPM series.

**Table 1:** Overview of *CTNNB1* variant analysis (exon 3) by Sanger sequencing (*ND*—not determined due to low amounts or decreased DNA quality of remaining sample DNA). Pathogenicity scores are according to the deep learning model AlphaMissense using AlphaFold for variant effect prediction [16]

No	*CTNNB1* variant (exon 3)	Single nucleotide variant	Pathogenicity score*	Variant class	ß-catenin immuno-histochemistry
A	ND	-	-	-	Positive
B	c.98G >C; p.Ser33Cys	Yes	0.989	Likely pathogenic	Negative
C	ND	-	-	-	Negative
D	ND	-	-	-	Negative
E	wildtype	-	-	-	Negative
F	c.101G˃A; pGly34Glu	Yes	1.000	Likely pathogenic	Negative

### Intranodal palisaded myofibroblastoma with amianthoid fibers show a unique clustering due to a specifically methylated gene signature

**Fig. 2:**
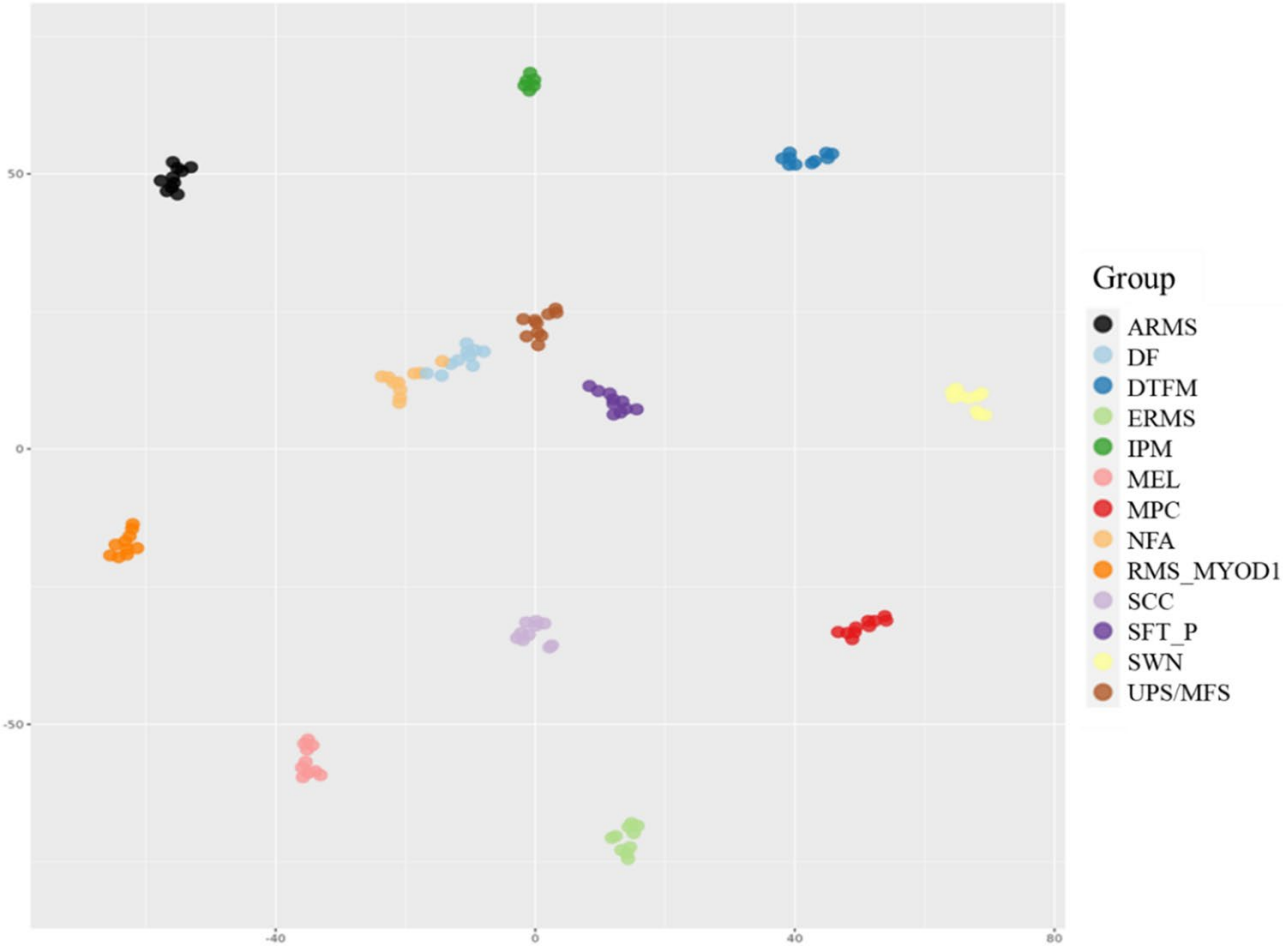
TSNE showing the distribution of intranodal palisaded myofibroblastoma (IPM) and 12 other tumor types. ARMS, alveolar rhabdomyosarcoma; DF, dermatofibroma; DTFM, desmoid type fibromatosis; ERMS, embryonal rhabdomyosarcoma; IPM, intranodal palisaded myofibroblastoma; MEL, melanoma; MPC, myopericytoma; NFA, nodular fasciitis; RMS-MYOD1, spindle cell rhabdomyosarcoma; SCC, squamous cell carcinoma; SFT-P, solitary fibrous tumor (peripheral); SWN, schwannoma; UPS/MFS, undifferentiated pleomorphic sarcoma/myxofibrosarcoma

UMAP plots of EpiDiP software demonstrated clustering of all analyzed IPM samples defining a selective and novel molecular tumor group, especially a respective methylation class (MC) (Figure S2). This cluster exhibits close molecular proximity to the methylome clusters of solitary fibrous tumors, sclerosing epithelioid fibrosarcomas, skeletal muscle inclusion body myositis, cavernomas, and other various sarcoma entities. UMAP plots created by the Heidelberg Classifier analysis also demonstrated a unique MC (data can be provided by the authors), demonstrating a close proximity to embryonal rhabdomyosarcomas, intramuscular myxomas, spindle cell rhabdomyosarcoma, and myofibromas as well as undifferentiated sarcomas with the utmost closest proximity of < 50 (two dimensional t-distributed stochastic neighbor embedding analysis (TSNE): x-axis: −100–0-100, y-axis: −100–0-100). Using other inputs to t-sne analysis, UMAP in Fig. 2 also demonstrates the unique MC (green dots) in comparison to histological mimics. Analyses of the CNV profiles (Heidelberg Classifier and EpiDip software) did not show chromosomal aberrations (Figure S1). Important tumor-associated genes such as *CCND1/2*, *CDK4/6*, *CDKNA/B*, *EGFR*, *FR3/TACC*, *GLI2*, *KIA1549*, *MDM2/4*, *MET*, *MGMT*, *MYB*, *MYCN*, *MYBL1*, *NF1*, *NF2*, *PPM1D*, *PTCH1*, *PTEN*, *SMARCB1*, *TERT*, *TP53*, and *RB1* did not show significant gains/amplifications or losses at higher resolution.

We additionally performed an in-house annotation analysis of the CNV profile. We used both human fibroblasts and human myoblasts, respectively, as the reference data set. Using this approach, we identified genes that showed the strongest gain or loss in all four tumor samples (A, B, E, and F) compared to the reference dataset (Tables S4 and S5; Figure S3).

We also plotted IMP MC together with potential mimics to demonstrate a good separation from those. Figure 2 demonstrates IPM forming a separate cluster different from tumors potentially mimicking this group such as alveolar rhabdomyosarcoma, dermatofibroma, desmoid type fibromatosis, embryonal rhabdomyosarcoma, melanoma, myopericytoma, nodular fasciitis, spindle cell rhabdomyosarcoma, squamous cell carcinoma, peripheral solitary fibrous tumor, schwannoma, and undifferentiated pleomorphic sarcoma/myxofibrosarcoma. Interestingly, other CTNNB1 ubiquitination motif-mutant tumors such as sinonasal glomangiopericytoma and nasopharyngeal angiofibroma show a high distance to IPM using TSNE analysis.

As a change of gene methylation might affect tumor pathogenesis, we analyzed the methylated genes of our series in more detail. Analysis of the 5-methylcytosine DNA methylation revealed differentially methylated genes (both hyper- and hypomethylated) in our sample when compared to the reference groups of myoblasts and fibroblasts. The following genes were more than 75-fold hypermethylated in all samples: *AMPD3*, *CACNA1D*, *CLASP1*, *FBXL13*, *HSPB3*, *KIRREL3*, *NEDD4*, *PDLIM3*, *PRKCE*, *PSD3*, *RUNX1*, *SCG5*, *SHROOM3*, *SLC8A1*, *SNAP47*, *ST6GALNAC5*, *TMEM176A*, *TTYH3*, and *ULK4*. Hypomethylated genes (17-fold) included *ADAMTS17*, *AGBL4*, *ARID5A*, *B3GALT4*, *BMP7*, *CABLES1*, *CALY*, *CAMKV*, *CCR10*, *DUSP2*, *FIS1*, *GLB1L2*, *GSTO2*, *KDF1*, *LEF1-AS1*, *MGC2889*, *HRASLS*, *MIB2*, *MKNK2*, *PNMAL2*, *RYR2*, *SH2B3*, *SLC38A10*, *SPEG*, *TRIM58*, *WSCD2*, and *ZNF835* (Table S6, Figures S4 and S5).

## Discussion

In our study, we were able to detect a unique epigenetic profile establishing a single methylation class of our series of intranodal palisaded myofibroblastoma with amianthoid fibers. This was demonstrated by specific clustering within UMAP plots created by the web application of EpiDiP as well as TSNE analysis using the Heidelberg classifier. This IPM MC was not visible prior to this study and will be included in the upcoming Heidelberg sarcoma.

Prior to our study, no comprehensive molecular study or methylation class has been established for these tumors without gross chromosomal genomic aberrations. We could refute the hypothesis that IPM could not be distinguished from other benign mesenchymal tumors by a specific molecular profile by demonstrating a unique methylation class of IPM. Herewith, we provide a tool for a molecular diagnosis, when differential diagnosis is needed beyond conventional pathology.

As those tumors show a myofibroblastic differentiation, we compared the methylation profile with the methylation profile of fibroblasts/myoblasts. We detected several strongly hypo- or hypermethylated genes that might be relevant for tumor development, such as genes involved in apoptosis, tumor suppression, NF-κB pathway regulation, immune system and inflammation response, metabolic processes, and stress response. These data need further reinforcement in a larger cohort to minimize artefacts due to batch effects or decreased DNA quality. To evaluate batch effects, it would be interesting to compare *CTNNB1*-mutant fibroblastic tumor types such as desmoid fibromatosis or nasopharyngeal angiofibroma that are out-of-batch for aberrant methylation of similar genes, but we are not aware of those data.

Concerning tumor formation and fiber deposition, *TRIM58* was one of the utmost hypomethylated genes in IPM. It is downregulated in tumors and important for ubiquitination [17, 18]. Disturbed ubiquitination could be one of the processes leading to a deposition of collagen with subsequent amianthoid fiber formation [19, 20]. The glutathione S transferases, which were upregulated in our investigation (*GSTT1*, *GSTTP1*), are involved in conjugation of asbestos fibers and might also play a role in amianthoid fiber formation in IPM [21]. Other genes of interest are hypermethylated genes such as *ARMC5*, *C3orf77*, and *OCA2* due to their important cellular functions [22].

We detected only some cases with a nuclear expression of ß-catenin or *CTNNB1* pathogenic variants of exon 3. To date, variants of exon 3 are reported in 71% of IPM, which is matching our finding of 75% [6]. Hotspot mutations in exon 3 of *CTNNB1* decreased the phosphorylation-dependent ubiquitination of β-catenin. In summary, these mutations frequently impact serine and threonine residues, which are the sites of phosphorylation by casein kinase 1 and glycogen synthase kinase 3. The process of ubiquitin-mediated degradation of β-catenin depends on phosphorylation at these specific sites. Consequently, mutations that modify these phospho-acceptor sites stabilize β-catenin, enabling its accumulation, translocation to the nucleus, and activation of WNT signaling. Missense variants in exon 3 were found in benign and malignant tumors. A nuclear expression has been correlated to *CTNNB1* variants in large studies [23]. *CTNNB1* is important for regulation of stem cell pluripotency, cancer signaling, and functions as an epithelial-mesenchymal transition-related gene. Mutations are common in mesenchymal tumors such as desmoid tumors, several benign and intermediate-biology neoplasms of soft tissue, in glomangiopericytoma, in solid tumors and carcinomas such as non-small cell lung carcinoma, colorectal, endometrial, pancreatic, and gastric carcinoma, craniopharyngeomas, medulloblastomas, and other neuroectodermal neoplasias, desmoid-type fibromatosis, melanomas, and other tumors [5]. Besides, those mutations were detected in superficial fibroma, a mesenchymal spindle cell tumor [24], and in sinonasal myxoma involving the ubiquitin recognition motif [25]. Thus, *CTNNB1* variants are relevant markers for IPM diagnosis; however, it does not suffice for an unambiguous molecular classification. In this study, there is an epigenetic proximity of IPM to desmoid-type fibromatosis, but not to sinonasal glomangiopericytoma, nasopharyngeal angiofibroma, and infantile sinonasal myxoma harboring variants in the ubiquitination motif [25–28]. Desmoid-type fibromatosis is epigenetically relatively close to IPM and typically harbors *CTNNB1* variants in exon 3. Interestingly, inactivation of the ß-catenin signaling via ubiquitination due to suppression by TRIM58 was reported in gastric cancer cells [29]. Further studies might elucidate if there is also a role in IPM.

Minor changes in the CNV plots were not assessed due to the decreased quality as we used FFPE samples and an increased unreliability of signal normalization on X and Y chromosomes, although recurrent amplification of *IL13RA2* on chromosome X might be of interest as it has recently been described to be a biomarker in glioma patients [30]. There are limitations of this study: the cohort is small and attributed to the rare occurrence and rare diagnosis of IPM. It therefore needs further collections to expand and strengthen the results. Interestingly, analysis of global methylation has become easier for FFPE tissue over the last years, allowing further studies to use the same assay for reanalysis. Novel methods such as long read nanopore sequencing are extremely promising but are still not sufficient for older FFPE samples and reduced DNA quality. Furthermore, signal normalization on X and Y chromosomes is still not reliable (personal communication with Juergen Hensch and Andreas von Deimling), which is the reason for not accessing genomic regions of X and Y chromosomes in our study. But relevant genes in these regions might be detected in further studies.

In conclusion, we demonstrated that IPM exhibits a characteristic and unique methylation profile. The clinical significance is based on the fact that in the case of a difficult differential diagnosis (e.g., very little material from punches that have no characteristic aspects), further molecular analyses are reliable and senseful. The increasing availability of methylome analysis in routine practice (e.g., using rapid nanopore sequencing) is a particular advantage here. The results show that histological mimics can be separated safely, emphasizing that these tumors are biologically different from other benign soft tissue tumors, even if the biological features are not yet fully understood. A molecular analysis intended to diagnostically confirm or exclude a lymph node metastasis can be very useful here.

## Supplementary Information

Below is the link to the electronic supplementary material.

Supplementary file1 (PDF 1643 KB)

## Data Availability

All data are available on request from first or senior authors.
