# Supplementary material for: Intranodal palisaded myofibroblastoma shows a unique epigenetic profile—first molecular study of their epigenetic and copy number variation profile
Source: Virchows Arch. 2025 Jul 18;487(6):1383–9. doi: 10.1007/s00428-025-04170-x (PMC12748110; doi:10.1007/s00428-025-04170-x)

Supplementary figures and tables

Table S1
Overview of the most important immunohistochemical results in all intranodal palisaded myofibroblastomas with amianthoid fibers investigated.

**No.**	**Years of age**	**Sex**	**α smooth muscle antigen**	**CD34**	**desmin**	**collagen type IV**	**S100**
A	67	female	+	-	-	ND	-
B	47	male	+	-	ND	ND	ND
C	42	male	+	ND	-	+	ND
D	67	male	+	ND	+ / -	ND	-
E	82	female	+	ND	ND	+	-
F	56	female	+	ND	+ / -	+ / -	ND

Table S2
List of antibodies used for immunohistochemical antibodies

**Antibody**	**Clone (or RTU)**	**Dilution (pretreatment)**	**Selling company**
anti-S100	RTU	(ER1, 20 min)	Agilent Technologies
anti-actin	HHF35	1:50 (ER1, 20 min)	Agilent Technologies
anti-CD31	JC70A	1:200 (ER1, 20 min)	Agilent Technologies
anti-CD34	QBEnd10	1:100 (ER1, 20 min)	Agilent Technologies
anti-AE1/AE3	RTU	(ER1, 20 min)	Agilent Technologies
anti Ki-67	MIB1	1:50 (ER1, 20 min)	Agilent Technologies
anti-D2-40	D2 40	1:3 (ER1, 20 min)	Agilent Technologies
anti-h-caldesmon	hCD	1:5 (ER1, 20 min)	Agilent Technologies
anti-EMA	E29	1:400 (ER1, 20 min)	Agilent Technologies
anti-neurofilament	2F11	1:50 (ER1, 20 min)	Agilent Technologies
anti-beta-catenin	Beta-Catenin 1	1:5 (ER1, 20 min)	Agilent Technologies
anti-CD117	Ckit	1:400 (ER1, 20 min)	Agilent Technologies
anti-ASMA	Asm-1	1:200 (ER1, 20 min)	Leica Biosystems
anti-desmin	DE-R-11, RTU	(ER1, 20 min)	Leica Biosystems
anti-SOX10	polyclonal	-	DCS-Diagnostics
anti-alpha-SMA	1A4	1:500 (buffer pH 9.0)	DAKO
anti-CD34	HPCA1	1:5000 (buffer pH 6.1)	Abcam
anti-desmin	D33	1:100 (buffer pH 9.0)	DAKO
anti-collagen IV	CIV22	1:100 (protein kinase, pH 6.1)	DAKO
anti-S100	polyclonal	1:2000 (buffer pH 6.1)	DAKO

Pretreatment with ER1 (20 min) was according to manufacturer's instructions.

Table S3
Primer used for amplifying *CTNNB1* exon 3

**Name of oligonucleotide**	**Sequence 5' to 3'**	**Annealing temperature**	**Alignment of *CTBNN1* exon 3 mRNA**
hCTNNB1fwd	TTTGATGGAGTTGGACATGG	54°C	232 to 430
hCTNNB1rev	CTGAGAAAATCCCTGTTCCC		
hCTNNB1fwd2	GGAACCAGACAGAAAAGCGG	56°C	256 to 455
hCTNNB1rev2	CAGCTACTTGTTCTTGAGTGAAGG		
hCTNNB1fwd3	CTGATTTGATGGAGTTGGACATGG	56°C	228 to 431
hCTNNB1rev3	ACTGAGAAAATCCCTGTTCCC		

Table S4
Genes with the highest gains in intranodal myofibroblastoma with amianthoid fibers (gain of > 0.4, $p < 0.05$ in all samples compared to the reference group of human fibroblasts* and human myoblasts** respectively).

**Gain of gene**	**Gene name**	**Gene function**
*AATK***	*Apoptosis Associated Tyrosine Kinase*	Serine/threonine-protein kinase, inhibits activation of the Na-K-Cl cotransporter, regulates **apoptosis**
*CTD-3080P12.3 / TERLR1***	*TERT Regulating LncRNA 1*	RNA gene, affiliated with the lncRNA class
*ENPP7***	*ectonucleotide pyrophospha-tase/phosphodiesterase*	activity against lipids, hydrolyzes sphingomyelin, antiproliferative, proapoptotic, competes with lysophospholipase D, inhibits PAF-induced inflammatory responses, **tumor suppressor**
*FAM167A-AS1 / c1orf90 ***	*FAM167A Antisense RNA 1*	RNA gene, affiliated with the lncRNA class, activating the noncanonical **NF-κB pathway**
*GSTT1***	*Glutathion S-Transferase T1*	catalyzes conjugation of reduced glutathione to electrophilic and hydrophobic compounds, diseases associated include **asbestosis**
*GSTTP1***	*Glutathione S-Transferase Theta 4*	enables glutathione transferase activity
*HLA-DQB1**	*Major Histocompatibility Complex, Class II, DQ Beta 1*	**immune system**, presenting peptides derived from extracellular proteins, expressed in antigen presenting cells such as B-lymphocytes, dendritic cells, and macrophages
*INPP5A**	*Type I inositol-1,4,5-trisphosphate 5-phosphatase*	mobilizes intracellular calcium, second messenger
*LAMTOR4***	*late endosomal/lysosomal adaptor, MAPK and MTOR activator 4*	guanyl-nucleotide exchange factor activity and molecular adaptor activity, response to amino acid stimulus, regulates TOR signalling, lysosomal
*MIR338***	*Micro RNA 338*	associated with lymphoma, squamous cell carcinoma
*MIR5694**	*Micro RNA 5694*	unknown
MIR657	*Micro RNA 657*	associated with type 2 diabetes and ovarian cancer
*PCCB**	*Propionyl-CoA Carboxylase Subunit Beta*	mitochondrial enzyme, catabolizes odd chain fatty acids, branched-chain amino acids isoleucine, threonine, methionine, valine and others
*TRIO**	*Trio Rho Guanine Nucleotide Exchange Factor*	required for neural crest cell migration, interacts with Dishevelled, cell adhesion, oncogenesis

*UPK3B***	*Uroplakin 3B*	role in AUM-cytoskeleton interaction in terminally differentiated urothelialium, formation of urothelial glycocalyx, role in preventing bacterial adherence
*VWA1**	*on Willebrand factor A domain-containing protein 1*	belongs to the von Willebrand factor, extracellular matrix protein, role in cartilage function

Table S5:
Genes with the highest losses in intranodal myofibroblastoma with amianthoid fibers (loss of > -0.4, $p < 0.05$ in all samples compared to the reference group of human fibroblasts* and human myoblasts** respectively).

**Gene loss**	**Gene name**	**Gene function**
*CCL4L2***	*C-C motif chemokine ligand 4 like 2*	induces chemotaxis of CCR5 or CCR1 expressing cells
*CD83***	*Cluster of Differentiation 83*	role in antigen presentation or the cellular inter-actions following lymphocyte activation
*CFP45**	*Cilia and flagella associated protein 45*	interacts with AK8 to create a cavity at the interface of the dimer that can accommodate AMP
CRAT37/***	*Cervical Cancer-Asso-ciated Transcript 37*	involved in cervical cancer and fibroblast microenvironment
*DCTN6***	*Dynactin subunit 6*	part of the dynactin complex activating motor dynein for ultra-processive transport along microtubules
*FABP3**	*Fatty acid binding protein 3*	thought to play a role in intracellular transport of long-chain fatty acids and acyl-CoA esters
*FHOD1***	*Formin homology 2 domain containing 1*	required for assembly of F-actin structures, depends on the Rho-ROCK cascade, coordinates microtubules, role in cell elongation, acts with ROCK1 for membrane blebbing
*GNAO1***	*G protein subunit alpha o1*	involved in various transmembrane signalling systems, stimulated by RGS14
*GP5***	*Glycoprotein V platelet*	mediates vWF-dependent platelet adhesion
*IDO2***	*Indoleamine 2,3-dioxygenase 2*	catalyses catabolism of tryptophan along kynurenine pathway, involved in immune regulation, role in tryptophan-related tumoral resistance
*KCNK3***	*Potassium Two Pore Domain Channel Subfamily K Member 3*	pH-dependent, voltage-insensitive, background potassium channel protein, acts as rectifier of potassium
*KRTAP19-6***	*Keratin associated protein 19-6*	intermediate filament embedded in interfilamentous matrix, consisting of hair keratin-associated proteins (KRTAP) essential for resistant hair shaft
*MIR548O2***	*MicroRNA 548o-2*	micro RNA gene
*NASP***	*Nuclear autoantigenic sperm protein*	required for DNA replication, cell cycle progression and proliferation, complex with HSP90 and H1 linker histones, stimulates HSP90 ATPase activity, locates to nucleus
*NFATC1***	*Nuclear factor of activated T cells 1*	role in inducible expression of cytokine genes in T-cells, controls expression in embryonic cardiac cells, regulates differentiation and death of T-cells and osteoclasts
*NUMB***	*NUMB endocytic adaptor protein*	regulates clathrin-mediated receptor endocytosis, role in neurogenesis (e.g. radial glial cells), mediates repair of brain ventricular wall damage
*NUP93***	*Nucleoporin 93*	role in nuclear pore complex assembly and/or main-tenance, anchors nucleoporins, regulates podocytes

*PLD5**	*Phospholipase D family member 5*	enables catalytic activity, predicted to be integral component of membrane
*PNPLA7***	*Patatin like phosphorlipa-se domain containing 7*	preferentially deacylates unsaturated lysopho-sphatidylcholines
*PKNOX1***	*PBX/knotted 1 homeobox 1*	activates transcription among PBX1A and HOXA1
*RBM20**	*RNA binding motif protein 20*	regulates mRNA splicing of genes encoding key structural proteins involved in cardiac development
*RERE***	*arginine-glutamic acid dipeptide repeats*	role as transcriptional repressor during development, role in cell survival, triggers caspase-3 activation and leads to cell death when overexpressed
*TULP4***	*TUB like protein 4*	substrate-recognition component of a Elongin-Cullin-SOCS-box protein E3 ubiquitin ligase complex to mediate ubiquitination and proteasomal degradation
VAV2/***	*Vav guanine nucleotide exchange factor 2*	role in angiogenesis, recruitment by phosphorylated EPHA2 is critical for EFNA1-induced RAC1 GTPase activation and endothelial cell migration/assembly
*WARS**	*tryptophanyl-tRNA synthetase 1*	aminoacylation activity, inhibits fluid shear stress-activated responses of endothelial cells, regulates ERK, Akt, and eNOS activation pathways associated with angiogenesis, cytoskeletal reorganization and shear stress-responsive gene expression

Table S6:

Hyper- and hypomethylated genes of myofibroblastoma in comparison to myoblasts and fibroblasts

Hypermethylation / CutOff >75	Hypomethylation / CutOff <17
AMPD3	*ADAMTS17*
CACNA1D	*AGBL4*
CLASP1	*ARID5A*
FBXL13	*B3GALT4*
HSPB3	*BMP7*
KIRREL3	*CABLES1*
NEDD4	*CALY*
PDLIM3	*CAMKV*
PRKCE	*CCR10*
PSD3	*DUSP2*
RUNX1	*FIS1*
SCG5	*GLB1L2*
SHROOM3	*GSTO2*
SLC8A1	*KDF1*
SNAP47	*LEF1-AS1*
ST6GALNAC5	*MGC2889;HRASLS*
TMEM176A	*MIB2*
TTYH3	*MKNK2*
ULK4	*PNMAL2*
	RYR2
	SH2B3
	SLC38A10
	SPEG
	TRIM58
	WSCD2
	ZNF835

Figure S1

CNV annotations of the six myofibroblastoma samples. The CNV profile was created with the Heidelberg brain classifier (version 12.8) and assessed on 13 March 2024 as well as with EpiDip (version 4.2 GPU), assessed on 13 March 2024).

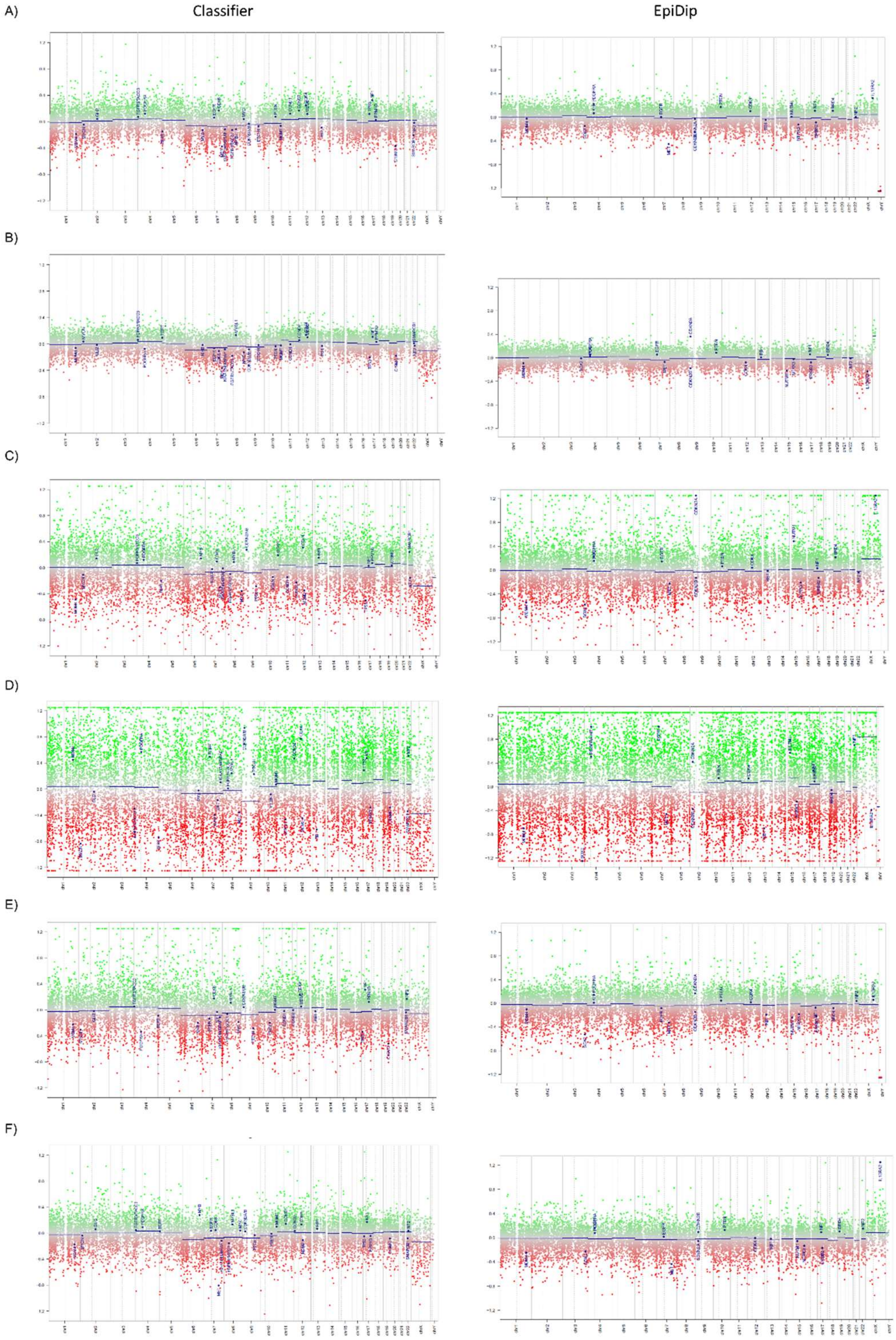

Figure S2

EpiDip analysis of the methylome detected by DNA methylation array analysis of our series of intranodal myofibroblastoma with amianthoid fibers. IDAT-files of the methylation analysis were uploaded to EpiDip server. UMAP plot was created with 25,000 probes and 800 px plot height. The last access was conducted on 20th March 2024.

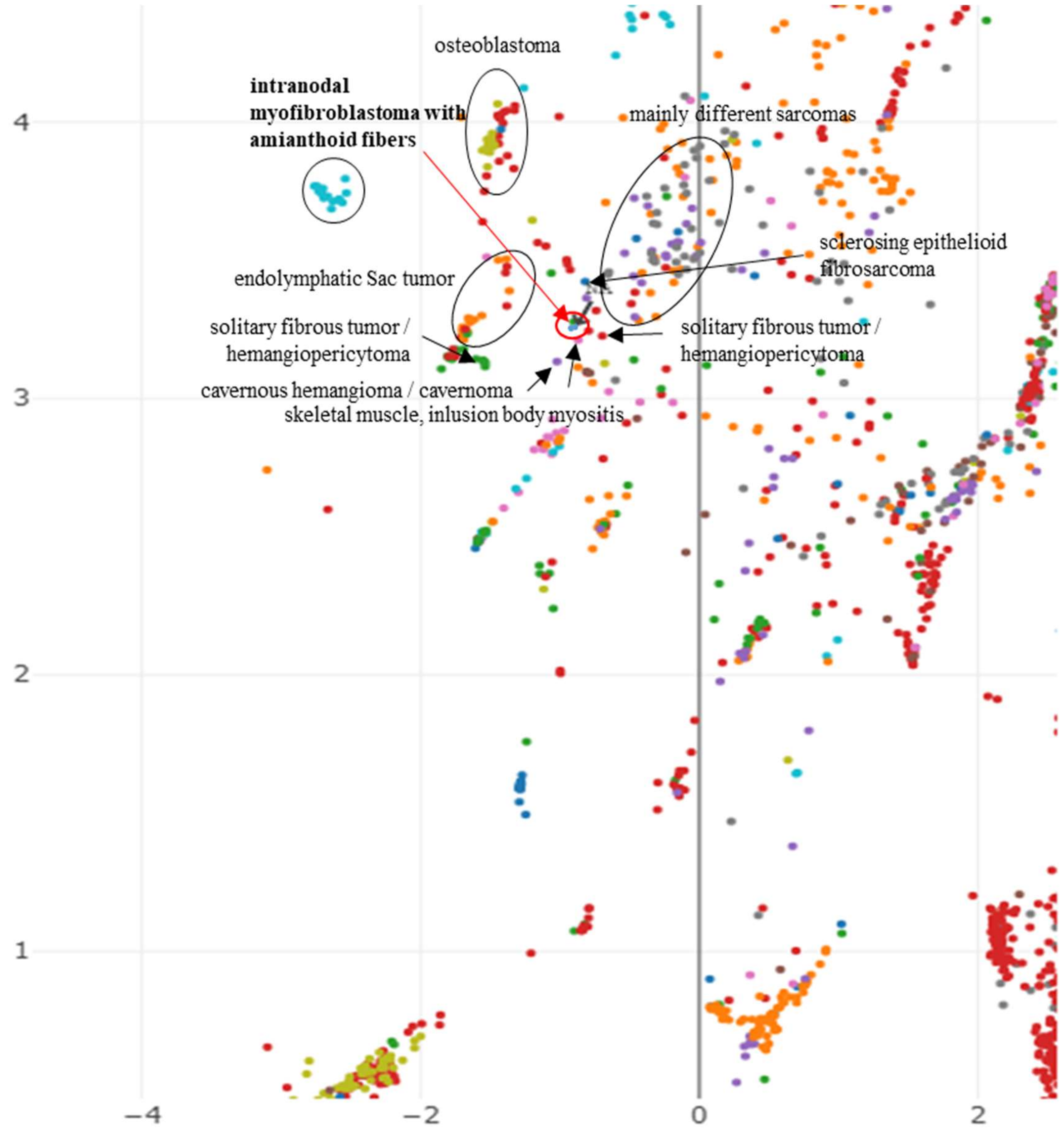

Figure S3

CNV profiles of the tumor samples (gains-green; losses-red).

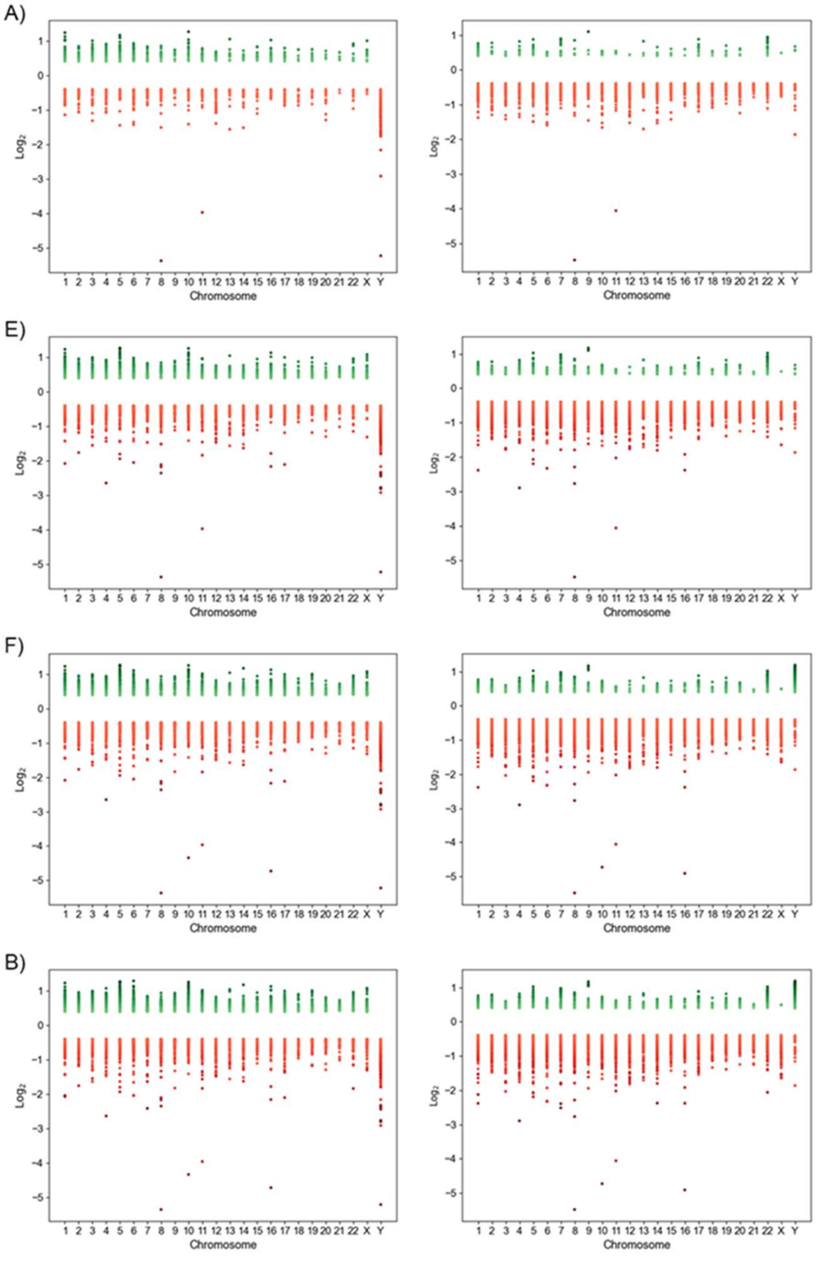

The IPM data were further analyzed to compare the tumour genome to a reference set of non-tumor cells using Python scripts (see material and methods). The only available non-tumour-associated reference datasets matching the cell type of IPM best in databases were those of human fibroblasts and myoblasts (also having been analyzed with the Infinium Methylation EPIC chip). After comparison we created Venn diagrams to address possible logical relations between our finite IPM collection and the normal cellular set and evaluated the overlap of all samples. The CNV is given (log2-ratio) for the comparison to myoblasts (left row) and to fibroblasts (right row) as reference groups (GSE213427) depicting chromosome on the x-axis. Sample C and D were excluded due to poor DNA quality. Tables S4 and S5 demonstrate the most up- and downregulated genes from this complex analysis.

Figure S4

Plotting of hypermethylation (cut off >75, $p < 0.05$). Hypermethylation was obtained as described in the method section using myoblasts (left row) and fibroblasts (right row) as reference group (GSE213427). Sample C and D were excluded due to poor DNA quality.

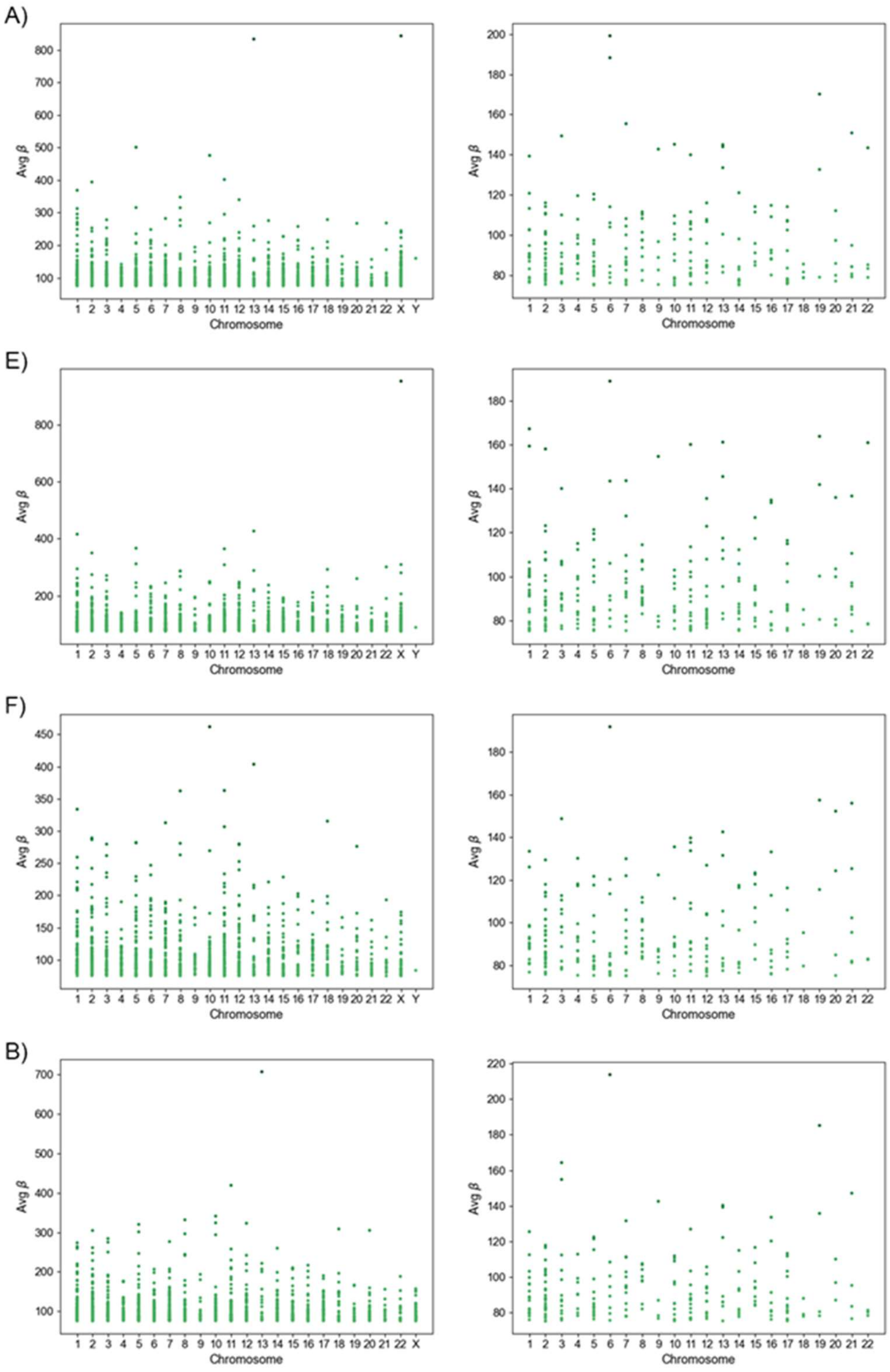

Figure S5

Hypomethylation (cut off <0.06, $p < 0.05$). Hypomethylation was obtained as described in the material and methods section using myoblasts (left row) and fibroblasts (right row) as reference group (GSE213427). Sample C and D were excluded due to poor DNA quality.

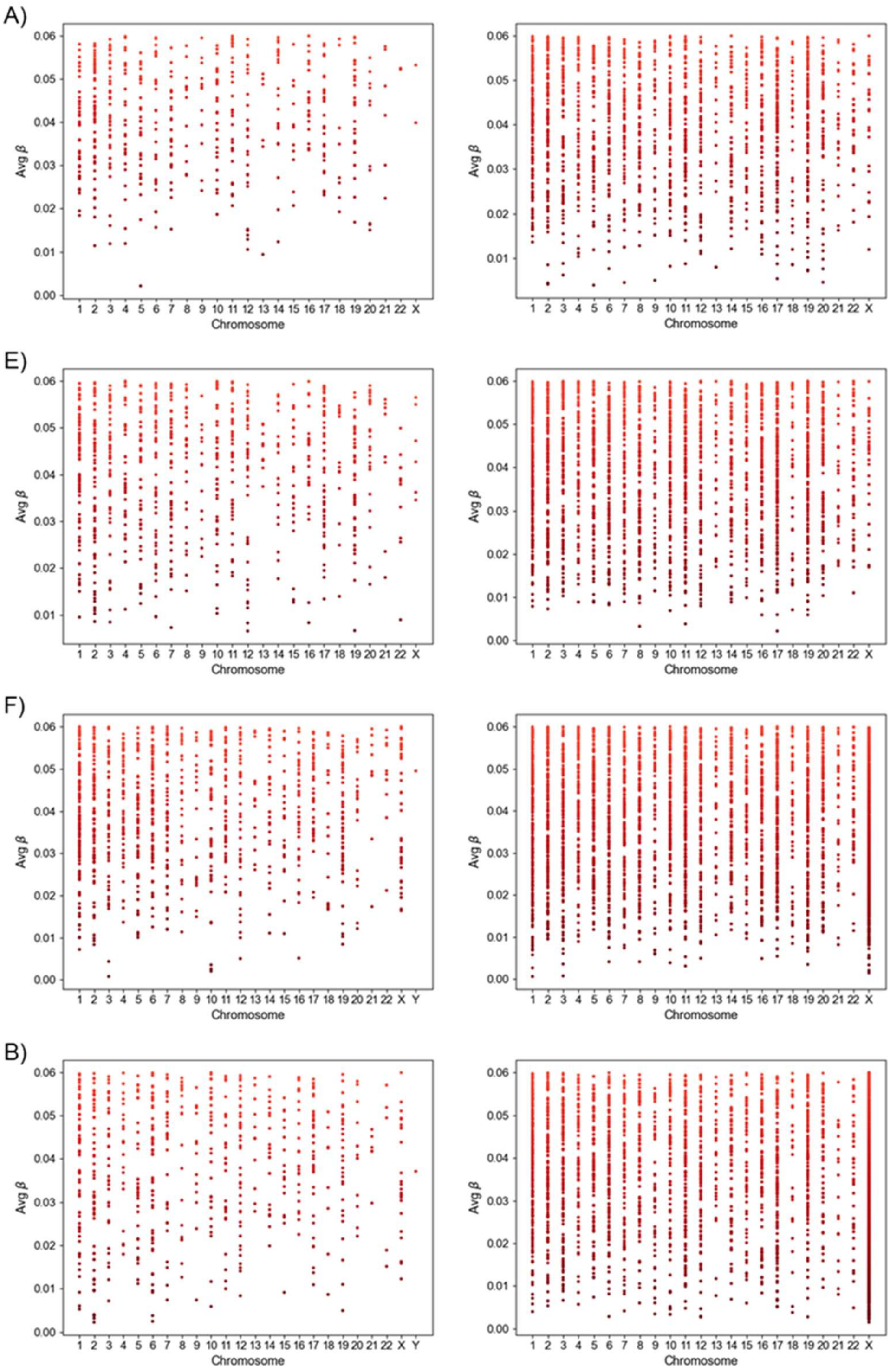

Supplement: Supplementary file 1 — Supplementary file1 (PDF 1643 KB) [file 428_2025_4170_MOESM1_ESM.pdf]
